# The fecal, oral, and skin microbiota of children with Chagas disease treated with benznidazole

**DOI:** 10.1371/journal.pone.0212593

**Published:** 2019-02-26

**Authors:** Carlos Robello, Doris Patricia Maldonado, Anna Hevia, Marina Hoashi, Paola Frattaroli, Valentina Montacutti, Adriana Heguy, Igor Dolgalev, Maricruz Mojica, Gregorio Iraola, Maria G. Dominguez-Bello

**Affiliations:** 1 Institut Pasteur de Montevideo, Montevideo, Uruguay; 2 Facultad de Medicina, Universidad de la República, Montevideo, Uruguay; 3 Universidad Andina Simón Bolívar, Sucre, Bolivia; 4 Division of Translational Medicine, New York University School of Medicine, New York, NY, United States of America; 5 University San Francisco Javier de Chuquisaca, Sucre, Bolivia; 6 Center for Integrative Biology, Universidad Mayor, Santiago de Chile, Chile; 7 Genome Technology Center, New York University Langone Medical Center, New York, NY, United States of America; Institute of Tropical Medicine (NEKKEN), Nagasaki University, JAPAN

## Abstract

**Background:**

Chagas disease is still prevalent in rural areas of South America. In endemic areas of Bolivia, school children are screened for the program of Chagas disease eradication of the Ministry of Health, and positive children are treated. Here, we compared the fecal, oral and skin microbiomes of children with or without Chagas disease, and before and after benznidazol treatment of infected children.

**Methods:**

A total of 543 Bolivian children (5–14 years old) were tested for Chagas disease, and 20 positive children were treated with Benznidazole. Fecal samples and oral and skin swabs were obtained before and after treatment, together with samples from a group of 35 uninfected controls. The 16S rRNA genes were sequenced and analyzed using QIIME to determine Alpha diversity differences and community distances, and linear discriminant analyses to determine marker taxa by infection status or treatment.

**Results:**

Twenty out of 543 children screened were seropositive for Chagas disease (3.7%) and were included in the study, together with 35 control children that were seronegative for the disease. Fecal samples, oral and skin swabs were taken at the beginning of the study and after the anti-protozoa therapy with Benznidazole to the chagasic children. Infected children had higher fecal *Firmicutes* (*Streptococcus*, *Roseburia*, *Butyrivibrio*, and *Blautia*), and lower *Bacteroides* and also showed some skin -but not oral- microbiota differences. Treatment eliminated the fecal microbiota differences from control children, increasing *Dialister* (class *Clostridia*) and members of the *Enterobacteriaceae*, and decreasing *Prevotella* and *Coprococcus*, with minor effects on the oral and skin bacterial diversity.

**Conclusions:**

The results of this study show differences in the fecal microbiota associated with Chagas disease in children, and also evidence that treatment normalizes fecal microbiota (makes it more similar to that in controls), but is associated with oral and skin microbiota differences from control children. Since microbiota impacts in children, it is important to determine the effect of drugs on the children microbiota, since dysbiosis could lead to physiological effects which might be avoidable with microbiota restoration interventions.

## Introduction

Chagas disease, also known as American Trypanosomaisis, is a neglected parasitic disease caused by the protozoan *Trypanosoma cruzi*, and transmitted to humans and animals via Triatomine insect vectors [1, 2]. *T*. *cruzi* is a stercoraria trypanosome that is deposited with the feces of the vector during a blood meal, and infection of the new host takes place when parasites penetrate through a skin lesion or by contact with mucous tissue (oral, nasal, conjunctivas); the parasite can be also transmitted by ingestion, vertical transmission or transfusion [1, 2].

It is estimated that infection with *T*. *cruzi* affects eight million people worldwide, with the majority of cases occurring in the Latin American countries where the parasite is endemic [1–4], and more recently Chagas disease has emerged in non-endemic regions such as the United States, Canada, Western Europe, Japan, and Australia, due to widespread immigration [5]. The clinical course of disease is usually divided into an acute and a chronic phase. After an incubation period of 2–3 weeks, the acute phase presents general mild and non-specific symptoms, that can include fever, inflammation at the inoculation site (chancre), unilateral palpebral edema, enlarged lymph nodes, and splenomegaly; this phase often passes unnoticed and symptoms resolve spontaneously in 4 to 8 weeks [1–3], after which most patients remain chronically infected, and approximately 30% of them develop chronic chagasic cardiomyopathy [6], where the apical aneurysm of the left ventricle constitutes the hallmark of the disease, and 10% gastrointestinal disease predominantly affecting the esophagus, colon, or both [3]. Although less frequent than cardiomyopathy, gastrointestinal Chagas disease has higher incidence in Bolivia, Chile and Paraguay than in the rest of South America, Central America and Mexico [7]. These geographical differences were thought to be the result of the genetic diversity of *T*. *cruzi* [8]. In fact, at least three major lineages (A, B and C) have been described in this parasite [9], and currently is accepted that there exist at least six discrete typing units (DTU) [10], and more recently *T*. *cruzi* isolates from bats were included as the seventh DTU. However, the analysis of circulating strains did not show significant association between DTUs and clinical manifestations of Chagas disease [1, 2, 11].

The only drugs proven effective against Chagas disease are Benznidazole (Bzn), formerly commercialized as Rochagan and Radanil (Roche), and Nifurtimox (Nfx), marketed as Lampit (Bayer). Both contain a nitro group linked to an imidazole or furan ring, respectively. Bzn has the best safety and efficacy profile, and is therefore the most used as first line treatment. Major limitations are the low potency of these drugs against parasites in the established chronic disease, which is the form most commonly encountered clinically, and unwanted side effects that lead to treatment discontinuation in some cases. The therapeutic benefit of Bzn in established mild to moderate Chagas disease has been under scrutiny in the Benznidazole Evaluation for Interrupting Trypanosomiasis (BENEFIT) trial [12], and up to date the mode of action of these drugs remains unknown.

It is well known the relevance of human microbiome, and how significant changes at its level (dysbiosis) can affect health, mainly as a consequence of the importance of microbial communities in immunological and biochemical functions. However the role of treatments on microbiome composition remains underexplored. In any case, it is clear that the effects of treatment should be disentangled from the effects of specific diseases on the human-associated microbiota [13], as is the case for the parasitic disease schistosomiasis, where either the parasite and the treatment produce significant differences in faecal microbiome [14]. It is noteworthy that Benznidazol has antibacterial action [15], but its effect on the microbiota remains unknown. In this study we compared the fecal, oral, and skin microbiota of children with or without Chagas, and the effect of Bnz therapy in infected children.

## Materials and methods

### Ethics

Ethical approval was received from the Ethical Committee of the SEDES (#001/2013), Departmental Health Service of Chuquisaca, Bolivia. Adult parents provided written informed consent on the child’s behalf, and children agreed verbally to participate. A total of 543 Bolivian children aged 5–15 years were screened for Chagas disease on a School in the Bolivian Qetchua town of Tarabuco, 63 Km SE of Sucre. A total of 20 children were seropositive for Chagas disease, and asymptomatic, and were recruited for the study and treated with Benznidazole by the local medical team (Program for Chagas Eradication, Bolivian Ministry of Health, following WHO protocols -WHO Chagas facts sheets, http://www.who.int/mediacentre/factsheets/fs340/en/-). A total of 35 Chagas disease negative children were matched as negative controls (**S1 Fig**).

### Diagnostic, treatment and sample collection

Diagnostic of Chagas disease was performed by immunocromatography (Chagas STAT-PAK Assay) and indirect hemagglutination test (HAI Chagas Polychaco). Positive children were treated with Benznidazol (5mg/kg/day) for 60 days. Negative children were not treated. Fecal, oral and skin samples were taken pre (day 0) and post (day 60)-treatment. In non-infected children, samples used as controls were taken at days 0 (pre) and 60 (post). Dry swabs were taken from the children’s oral mucosa, skin (volar arm) and children provided stool samples in appropriate collectors. A total of 20 Chagas disease positive and 35 negative children provided samples at the time before and after positive children has their treatment. Samples were frozen at -80 Celsius until processing.

### 16S rRNA gene sequencing and analyses

DNA was extracted from the samples using the MoBio PowerSoil kit. The region V3-V4 of the 16S rRNA gene was amplified as described previously [16], and amplicons were purified and sequenced using the Illumina MiSeq platform. The QIIME (version 1.8.0) pipeline was used to process and analyze 16S rRNA sequences [17], estimate bacterial diversity and compare bacterial communities between the children groups. Forward and reverse reads were joined using fastq-join specifying a minimum of 10 overlapping base pairs and 20% maximum allowed differences within overlap regions. A total of 2,719,027 sequences (mean of 10,662 sequences/sample) were retained after quality filtering, using default parameters except for a Phred score cutoff of >19 and a maximum allowance of 3 N characters. Operational taxonomic units (OTUs) were picked through a closed-reference protocol with uclust [18] against the GreenGenes [19] 13_8 reference database at the 97% identity level, aligned using PyNAST [20]. Taxonomic assignments were made using RDP Classifier 2.2 [21]. Beta diversity calculations were done using unweighted UniFrac [22] and visualized through Emperor [23], R (3.2.1) and phyloSeq (1.12.2). Taxa that differentiated infected and non-infected children and microbiota before and after treatment were identified using the biomarker discovery algorithm LEfSe, version 1.0 [24] with LDA scores > 3.0. Only taxa with a minimum mean relative abundance of 0.5% were retained for further analysis. Raw sequencing reads were uploaded to the public database Qiita (Study ID # 11724) and to the European Bioinformatics Institut (EBI) database under accession number ERP113722.

## Results

We performed fecal microbiome analyses on 55 children, 20 that resulted Chagas positive, and 35 age matched controls. Positive children (but not control population) were treated with benznidazol.

### Fecal, oral and skin microbiome and Chagas disease

**Fig 1:**
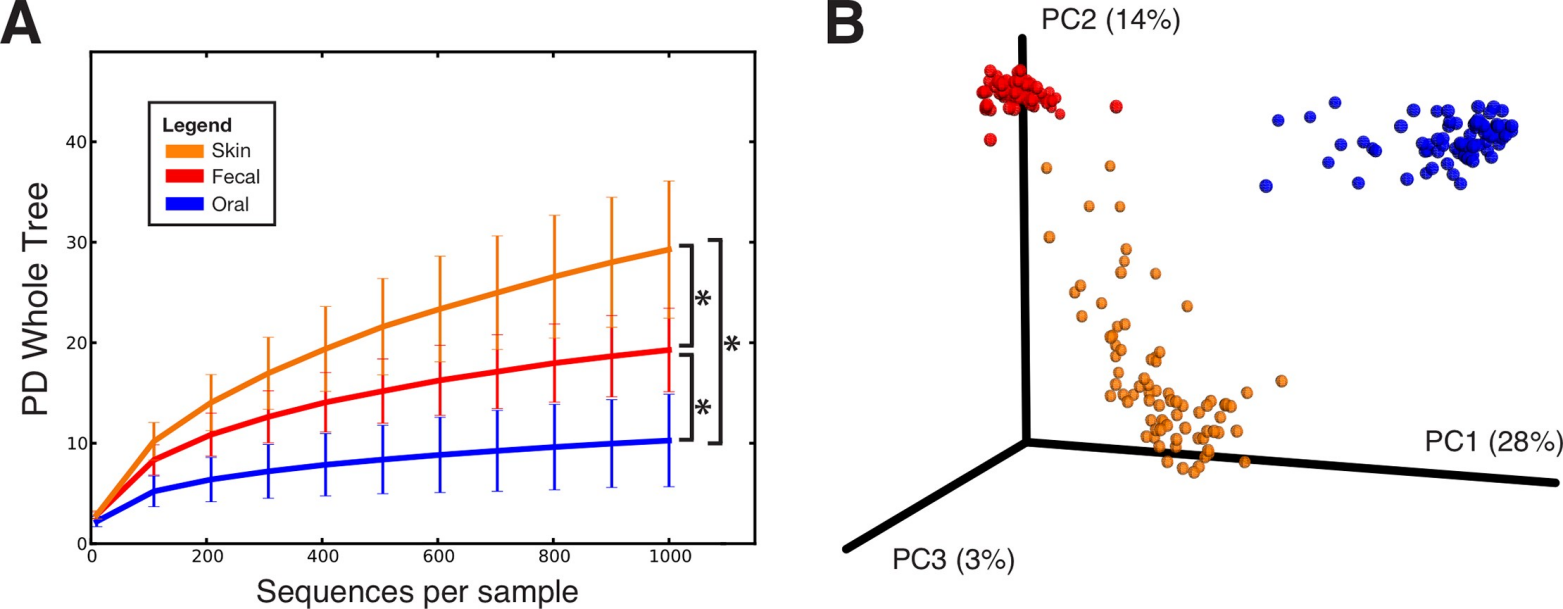
Alpha and Beta diversity of combined fecal, oral, and skin samples from all individuals. (A) Rarefaction curves using Faith’s Phylogenetic Diversity (PD Whole Tree) metric at 1000 sequences per sample (* p<0.01, t-test) (B) PCoA plot using the unweighted UniFrac metric shows that samples cluster first by body site.

**Fig 2:**
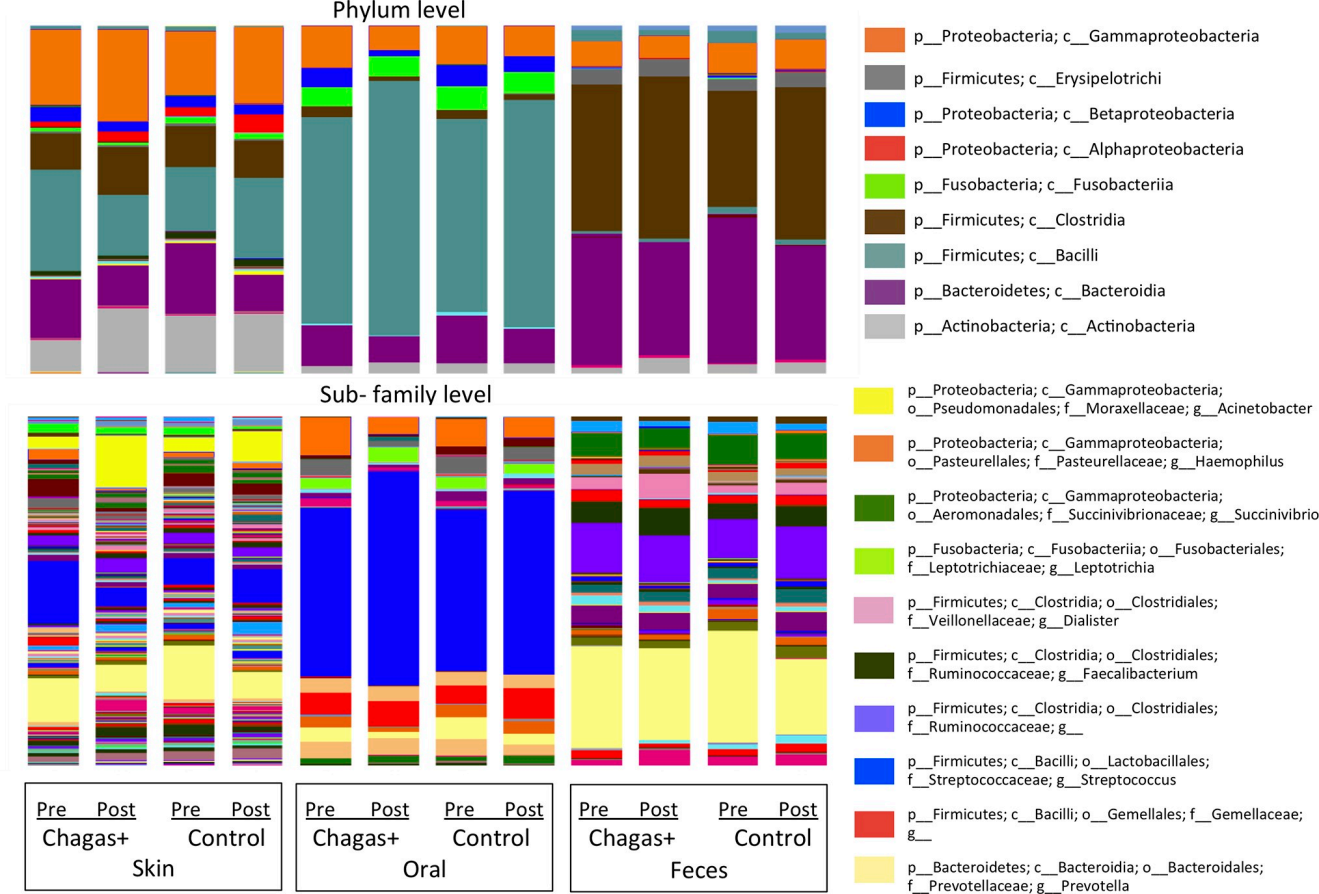
Relative bacterial taxa abundances in body sites of children with and without Chagas disease both before and after the treatment time point. Taxa are shown at the (A) phylum level and (B) the lowest classified level, and the labels below the plots indicate infection status (Chagas+ or control) as well as the time point (“Pre” = before treatment, “Post” = post-treatment). A taxa name ending in “g__” indicates that the OTU was not identified at the genus level. Sequences were rarefied at 1000 sequences/sample.

The microbiota differed by body site, as expected (**Fig 1**). Rarefaction curves of bacterial species diversity by body site, showed that the skin had the highest diversity, followed by fecal and oral. There were no significant differences by gender. The most abundant arm skin taxa were *Prevotella* (15.2%) and *Streptococcus* (9.7%; **Fig 2**). The fecal microbiota was dominated by *Prevotella* (30.2%), *Ruminococcaceae* (9.8%) and *Succinivibrio* (7.1%) (**Fig 2**). The oral microbiota was dominated by *Streptococcus* (45%), *Prevotella (7*.*2%)*, *Haemophilus* (6.8%), and members of the family *Gemellaceae* (5%), and there were no differences by infection status (**Fig 2**).

**Fig 3:**
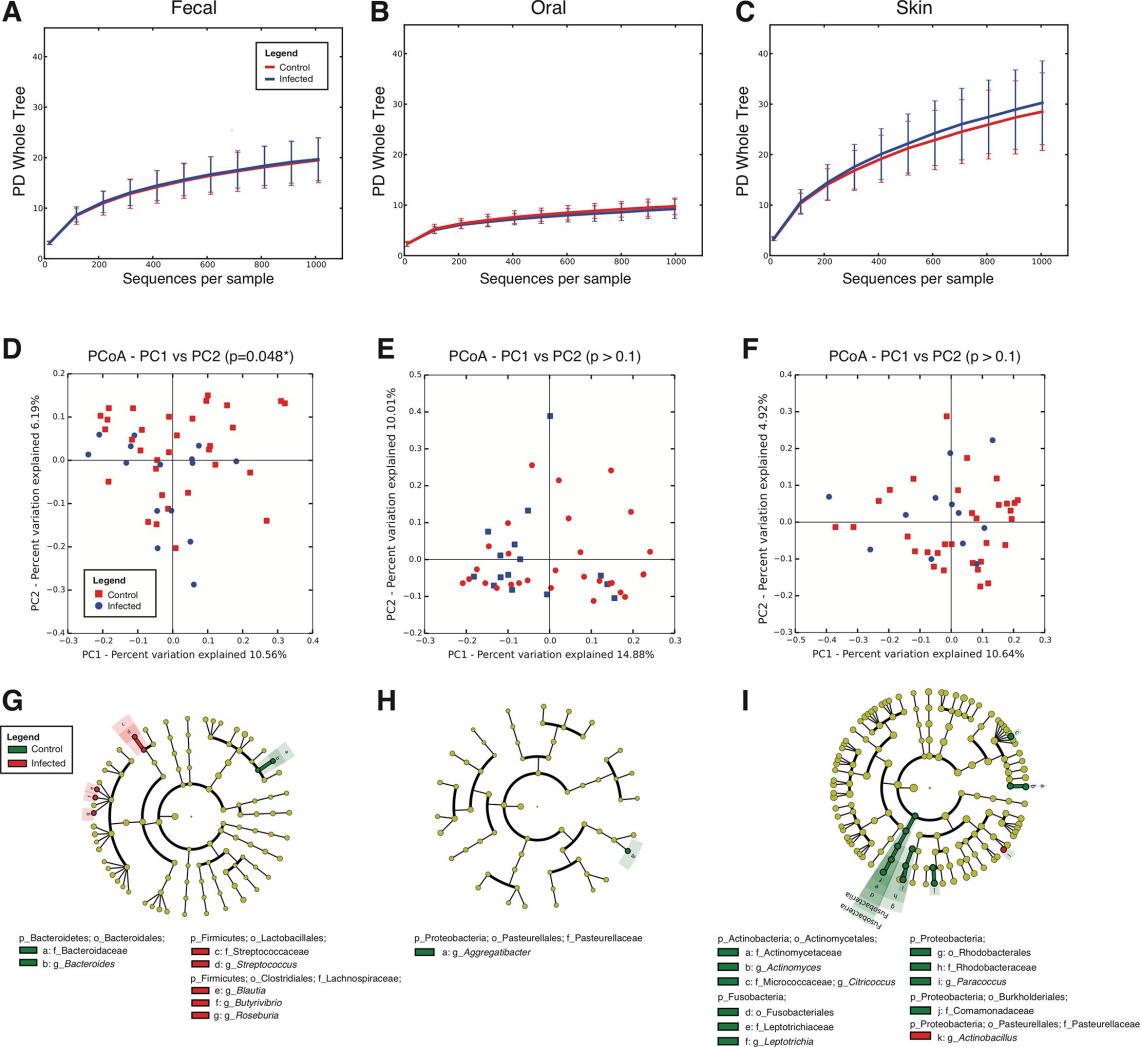
Effect of Chagas disease infection status on Alpha and Beta diversity of the microbiota in different body sites. (A-C) Rarefaction curves for fecal, oral, and skin samples infected (respectively 15, 15 and 12 samples) and uninfected children (respectively 32, 29 and 32 samples) at the initial time point, using Faith’s Phylogenetic Diversity (PD Whole Tree) metric at 1000 sequences per sample. (D-F). PCoA plots for fecal, oral, and skin samples using the unweighted UniFrac metric. (G-I) Taxa that best differentiate between Chagas disease seropositive and seronegative (control) children using LEfSe (LDA score cutoff of 3.0 and a minimum mean abundance of 0.1%).

After screening, a total of 20 children seropositive for Chagas disease were studied, whereas 35 samples from seronegative children were used as control population. Although this group was not treated with benznidazol, samples were taken at days 0 and 60, and named "pre" and "post" respectively. We then compared infected and non-infected children using Faith’s Phylogenetic Diversity (PD_whole_tree), and no differences were observed in Alpha diversity (**Fig 3A–3C**). However, when comparing Beta diversities using the UniFrac distance marginal differences were evidenced in fecal communities (**Fig 3D–3F**; *p* = 0.048, Permanova test). Infected children showed increased fecal *Streptococcus*, *Blautia*, *Butyrivibrio* and *Roseburia* and lower fecal *Bacteroides* (**Fig 3G**). Also, infected children presented increased skin *Actinobacillus*, and decreased *Actinobacteria* (*Actinomyces* and *Citricoccus)*, *Leptotrichia*, *Paracoccus*, and *Comamonadaceae*, and no differences in oral bacteria were found between infected children and controls (**Fig 3H and 3I**).

### Effect of treatment on the microbiota of infected children

**Fig 4:**
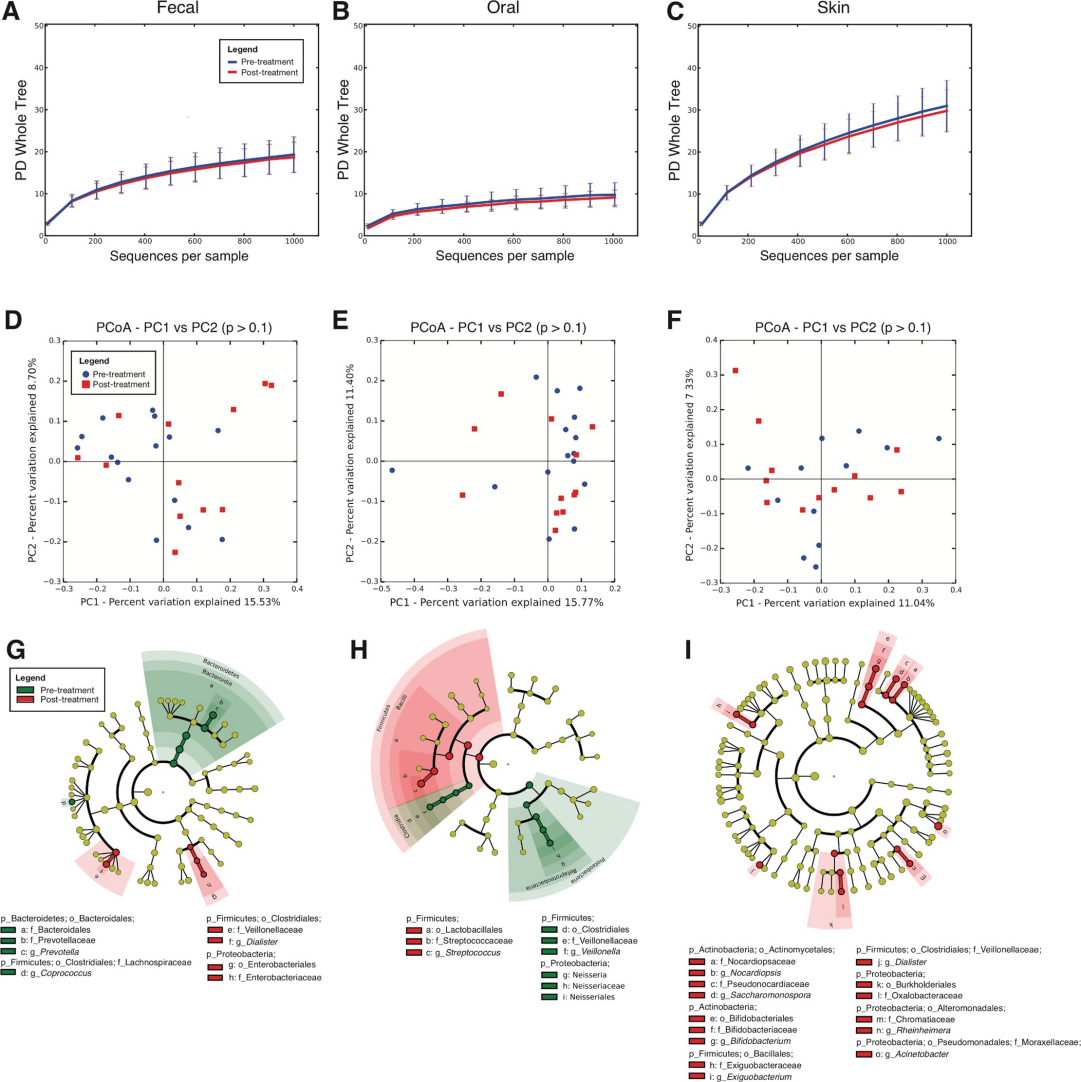
Effect of treatment on the microbiota of infected children. (A-C) Rarefaction curves for fecal, oral, and skin samples of infected children pre-treatment (respectively 15, 12 and 12 samples) and post treatment (respectively 12in each body site), using Faith’s Phylogenetic Diversity (PD Whole Tree), rarefied at 1000 sequences per sample (D-F) PCoA plots for fecal, oral, and skin samples using the unweighted UniFrac metric. (G-I) Taxa that best differentiate between children before and after treatment using LEfSe (LDA score cutoff of 3.0 and a minimum mean abundance of 0.1%).

We then compared the microbiota of infected children before and after the treatment with Bzn, and neither Alpha nor Beta diversity changed significantly (**Fig 4A–4F and S2 Fig**), whereas Beta diversity differed in the fecal, oral and skin microbiota. After the treatment, the fecal microbiota had reduced *Prevotella* and *Coprococcus* (*Clostridia*), and increased *Dialister* and *Enterobacteriaceae* (**Fig 4G**); the oral microbiota decreased Veillonella and *Neisseria*, and increased *Streptococcus* (**Fig 4H**); and the skin had increased *Bifidobacterium*, *Saccharomonospora* and Nocardiopsis, and increased *Firmicutes* (*Exiguobacterium* and *Dialister*) and *Proteobacteria* (*Acinetobacter*, *Rheinheimera*, and *Oxalobacteraceae*) (**Fig 4I**). Children in the control group did not change significantly their microbiota Alpha or Beta diversities over time, and some populations changed in different ways to those in treated children (**S3 and S4 Figs**). Their decreased oral Shannon diversity index and also decreased over time (**S2 Fig**).

## Discussion

Chagas disease constitutes a major concern for public health in Latin America, and more recently has emerged in non-endemic regions such as the Western Europe, United States, Canada, Japan and Australia, due to widespread immigration. The nitroimidazole Bnz constitutes the first line treatment, and its efficacy has been demonstrated in children [25]. Moreover, recently the U.S. Food and Drug Administration (FDA) granted accelerated approval to Bnz for use in 2 to 12 year-old children with Chagas disease, which constituted the first drug approved in the United States for the treatment of this disease. Although its mode of action remains unclear, its pleiotropic effects are well known and can be explained by the large amount of their derived metabolites that form adducts with functionally relevant biomolecules [26]. In this work we found differences in the microbiota of children infected with *T*. *cruzi*, and also differences associated with treatment. The differences in fecal microbiota associated to Chagas disease in children disappear with the treatment, suggesting that the effect of the infection on the gut microbiota might be more important than the effect of the treatment. Despite the age and diet effect on the variations in the fecal microbiota, there were statistically significant variation between children that were Chagas positive or not, and of Chagas infected and treated children in relation to their microbiota before treatment. The immune response against *T*. *cruzi* might be responsible, at least in part, of these changes, but this deserves further study.

We found differences in fecal, skin and oral microbiota associated to the Bnz treatment, which could be a direct consequence of the antibacterial activity of Bnz. In fact, its biotransformation produces several covalent thiol adducts resulting in the depletion of glutathione, trypanothion and cysteine in the parasite, with the consecutive increase in sensitivity to oxidants [26] and, in addition, Bnz covalently interacts with nucleic acids, proteins and lipids [27], which may also account for its activity on microbiota composition. Remarkably, Bnz is a pro-drug without activity and its oral ingestion not necessarily causes antibacterial effects. By the contrary, Bnz needs to be activated and in *T*. *cruzi* the nitroreductases NTR I and *Tc*OYE are the main responsible for this activation [28–29]. Interestingly, both nitroreductases belong to highly conserved families in prokaryotes, and even a bacterial origin through horizontal transfer has been postulated for *Tc*OYE [29]. We therefore postulate that activation of Bnz could account immediately after ingestion by bacterial nitroreductases, although it deserves to be studied in detail.

As mentioned, Bnz has several undesirable side effects, being the most notorious skin manifestations like hypersensitivity and dermatitis with cutaneous eruptions, which have been classically attributed to a direct effect on skin [28]. However the results found here on skin microbiota can also explain, at least in part, these skin effects. Finally, we cannot exclude the possibility that host response to treatment, because of its high toxicity, can also affect microbiota. Future studies, including metatranscriptomics and metabolomics, should add important mechanistic information on the effect of infection and of treatment on the microbiome and host immune responses.

In summary, human Chagas disease was traditionally analyzed as a binomial host-parasite interaction, and we studied here a third variable that is the affection on host microbiota both through the parasite by itself, and through the effects of Bnz. Considering this, together with previous reports in animal models showing that early impacts on the microbiota lead to physiological effects [30–33], understanding the effects of child infections and treatment drugs on the microbiota emerges as a new target to optimize Chagas disease treatment strategies.

## Supporting information

S1 Checklist STROBE checklist. (PDF)

S1 Fig Experimental design for sampling Chagas disease positive children followed until treatment was completed. (PDF)

S2 Fig Shannon Alpha diversity differences by Chagas disease status and treatment. Rarefaction curves for fecal, oral, and skin samples at 1000 sequences per sample are shown, comparing (A-C) infected and control groups, (D-F) pre- and post-treatment groups, and (G-I) days 0 (blue) and 60 (red). Oral diversity significantly (*p<0.05, nonparametric t-test) decreased at the post-treatment time point in both control and infected groups).
(PDF)

S3 Fig Richness differences by infectious status and treatment. Rarefaction curves for fecal, oral, and skin samples based on the number of unique OTUs at 1000 sequences per sample are shown, comparing (A-C) Chagas infected and control groups, (D-F) pre- and post-treatment groups, and (G-I) days 0 (blue) and 60 (red). No significant (p < 0.05) differences between groups were found.
(PDF)

S4 Fig Temporal effects on Alpha and Beta diversity of the microbiota in different body sites in control children. (A-C) Rarefaction curves for fecal, oral, and skin samples using Faith’s Phylogenetic Diversity (PD Whole Tree) metric at 1000 sequences per sample. (D-F) PCoA plots for fecal, oral, and skin samples using the unweighted UniFrac metric. (G-I) Taxa that best differentiate between children at the time points before (n = 32 for fecal and skin, and 29 for oral) and after treatment (n = 22 for fecal and skin, 21 for oral) found using using LEfSe using an LDA score cutoff of 3.0 and a minimum mean abundance of 0.1%.
(PDF)
