# Correction: The fecal, oral, and skin microbiota of children with Chagas disease treated with benznidazole

**DOI:** 10.1371/journal.pone.0215199

**Published:** 2019-04-04

**Authors:** Carlos Robello, Doris Patricia Maldonado, Anna Hevia, Marina Hoashi, Paola Frattaroli, Valentina Montacutti, Adriana Heguy, Igor Dolgalev, Maricruz Mojica, Gregorio Iraola, Maria G. Dominguez-Bello

There are errors in the author affiliations. The correct affiliations are:

Carlos Robello^1,2^, Doris Patricia Maldonado^3^, Anna Hevia^4^, Marina Hoashi^4^, Paola Frattaroli^4^, Valentina Montacutti^4^, Adriana Heguy^5^, Igor Dolgalev^5^, Maricruz Mojica^6^, Gregorio Iraola^1,7^, Maria G. Dominguez-Bello^4,8^

**1** Institut Pasteur de Montevideo, Montevideo, Uruguay, **2** Facultad de Medicina, Universidad de la República, Uruguay, **3** Universidad Andina Simón Bolívar, Sucre, Bolivia, **4** Division of Translational Medicine, New York University School of Medicine, New York, NY, United States of America, **5** Genome Technology Center, New York University Langone Medical Center, New York, NY, United States of America, **6** University San Francisco Javier de Chuquisaca, Sucre, Bolivia, **7** Center for Integrative Biology, Universidad Mayor, Santiago de Chile, Chile, **8** Department of Biochemistry and Microbiology, Rutgers University, New Brunswick, NJ, United States of America.
