# Supplementary material for: The fecal, oral, and skin microbiota of children with Chagas disease treated with benznidazole
Source: PLoS One. 2019 Feb 26;14(2):e0212593. doi: 10.1371/journal.pone.0212593 (PMC6391005; doi:10.1371/journal.pone.0212593)
Supplement: S1 Checklist — (PDF) [file pone.0212593.s001.pdf]

STROBE Statement—Checklist of items that should be included in reports of ***case-control studies***

	Item No	Recommendation
**Title and abstract**	1	(*a*) Indicate the study's design with a commonly used term in the title or the abstract **Done. Abstract, paragraph 2.**
		(*b*) Provide in the abstract an informative and balanced summary of what was done and what was found **Done in the Abstract**
**Introduction**		
Background/rationale	2	Explain the scientific background and rationale for the investigation being reported **Done. background, lines 42-46.**
Objectives	3	State specific objectives, including any prespecified hypotheses **Done. Lines 72-83, Author Summary.**
**Methods**		
Study design	4	Present key elements of study design early in the paper **Done. Methods, paragraph 2, "Sample collection".**
Setting	5	Describe the setting, locations, and relevant dates, including periods of recruitment, exposure, follow-up, and data collection **Done, methods, Paragraph 1.**
Participants	6	(*a*) Give the eligibility criteria, and the sources and methods of case ascertainment and control selection. Give the rationale for the choice of cases and controls **Done, methods, Paragraph 1.**
		(*b*) For matched studies, give matching criteria and the number of controls per case **Done**
Variables	7	Clearly define all outcomes, exposures, predictors, potential confounders, and effect modifiers. Give diagnostic criteria, if applicable **Done, methods, Paragraph 2.**
Data sources/ measurement	8*	For each variable of interest, give sources of data and details of methods of assessment (measurement). Describe comparability of assessment methods if there is more than one group **Done, methods, Paragraph 3, "16S rRNA gene sequencing and analyses".**
Bias	9	Describe any efforts to address potential sources of bias **Done, Methods, Paragraph 3, "16S rRNA gene sequencing and analyses".**
Study size	10	Explain how the study size was arrived at **Done, Methods, Paragraph 1, "Ethics".**
Quantitative variables	11	Explain how quantitative variables were handled in the analyses. If applicable, describe which groupings were chosen and why **Done, Methods, Paragraph 2**
Statistical methods	12	(*a*) Describe all statistical methods, including those used to control for confounding **Done**
		(*b*) Describe any methods used to examine subgroups and interactions **Done, Methods, Paragraph 2**
		(*c*) Explain how missing data were addressed **We did not have missing data**
		(*d*) If applicable, explain how matching of cases and controls was addressed **Not applicable**
		(*e*) Describe any sensitivity analyses

		**Done, Methods, Paragraph 3, "16S rRNA gene sequencing and analyses".**
**Results**		
Participants	13*	(a) Report numbers of individuals at each stage of study—eg numbers potentially eligible, examined for eligibility, confirmed eligible, included in the study, completing follow-up, and analysed<br>**Done. Results, paragraph 2.**
		(b) Give reasons for non-participation at each stage<br>**Not applicable.**
		(c) Consider use of a flow diagram<br>**Done. Supplementary Figure 1.**
Descriptive data	14*	(a) Give characteristics of study participants (eg demographic, clinical, social) and information on exposures and potential confounders<br>**Done**
		(b) Indicate number of participants with missing data for each variable of interest<br>**Done**
Outcome data	15*	Report numbers in each exposure category, or summary measures of exposure<br>**Done**
Main results	16	(*a*) Give unadjusted estimates and, if applicable, confounder-adjusted estimates and their precision (eg, 95% confidence interval). Make clear which confounders were adjusted for and why they were included<br>**Done, results, Paragraph 2 and 4.**
		(*b*) Report category boundaries when continuous variables were categorized<br>**Not applicable.**
		(*c*) If relevant, consider translating estimates of relative risk into absolute risk for a meaningful time period<br>**Not applicable.**

Other analyses	17	Report other analyses done—eg analyses of subgroups and interactions, and sensitivity analyses<br>**Not applicable.**
**Discussion**		
Key results	18	Summarise key results with reference to study objectives<br>**Done. Discussion, paragraph 1.**
Limitations	19	Discuss limitations of the study, taking into account sources of potential bias or imprecision. Discuss both direction and magnitude of any potential bias<br>**Done. Discussion, paragraph 3.**
Interpretation	20	Give a cautious overall interpretation of results considering objectives, limitations, multiplicity of analyses, results from similar studies, and other relevant evidence<br>**Done along the Discussion section.**
Generalisability	21	Discuss the generalisability (external validity) of the study results<br>**Done in Discussion, paragraph 4.**
**Other information**		
Funding	22	Give the source of funding and the role of the funders for the present study and, if applicable, for the original study on which the present article is based<br>**Done in the "Financial Disclosure" section**
