# Supplementary figures and images for: The fecal, oral, and skin microbiota of children with Chagas disease treated with benznidazole
Source: PLoS One. 2019 Feb 26;14(2):e0212593. doi: 10.1371/journal.pone.0212593 (PMC6391005; doi:10.1371/journal.pone.0212593)

Fig S1. Experimental design, Chagas study

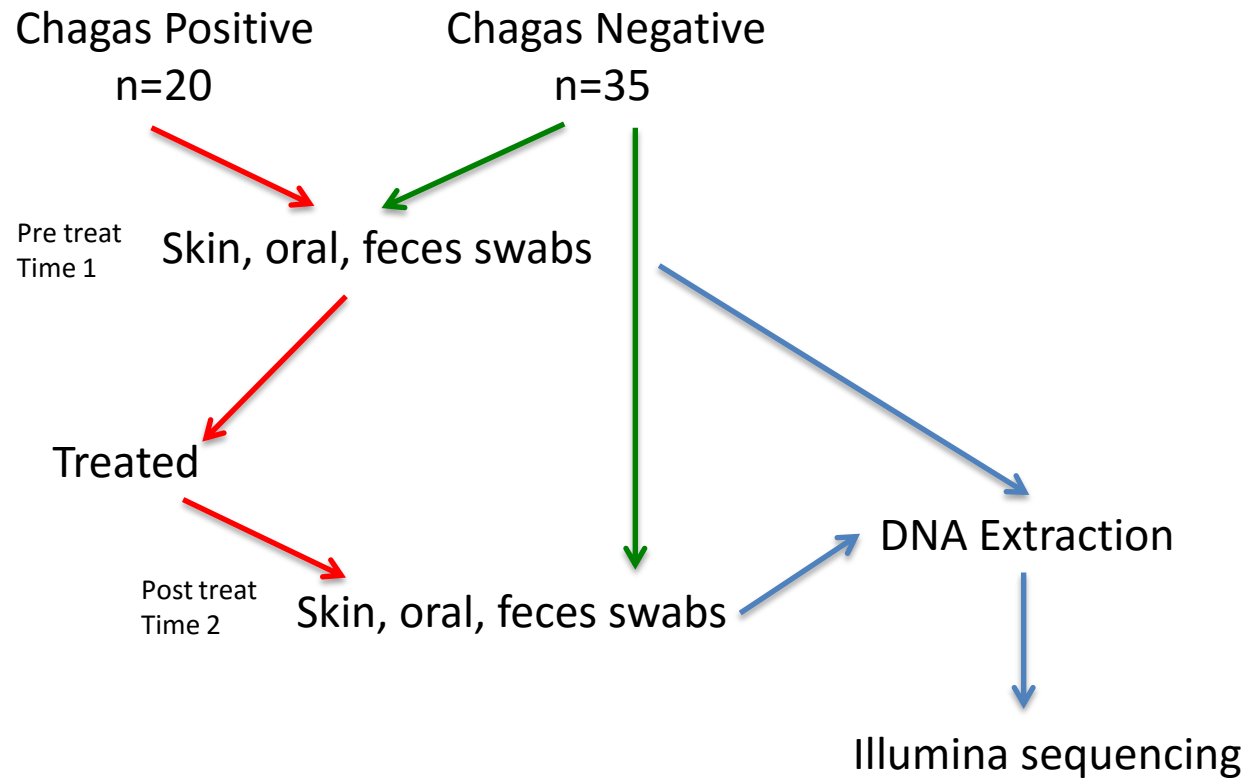

Supplement: S1 Fig — (PDF) [file pone.0212593.s002.pdf]

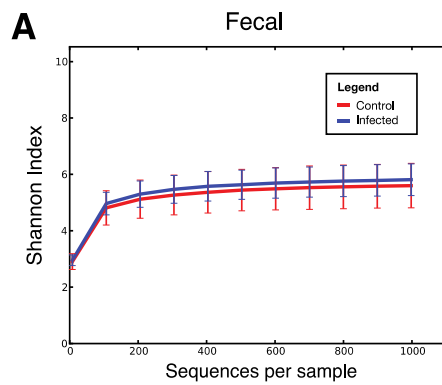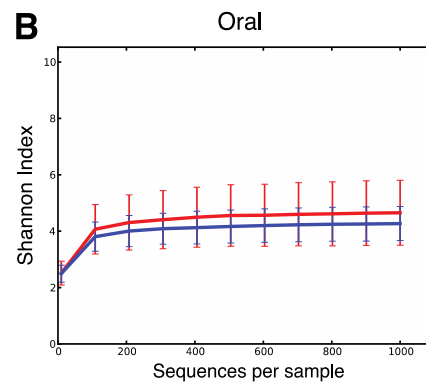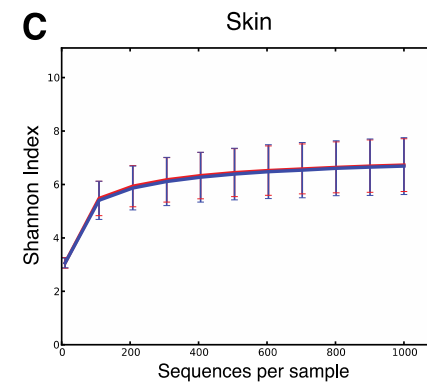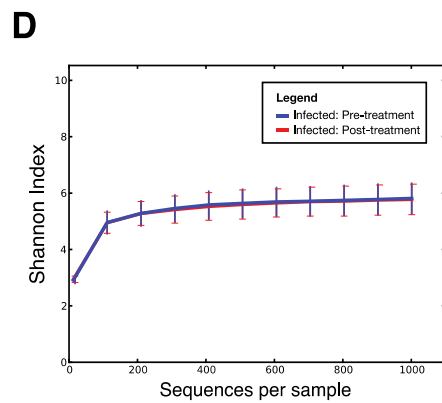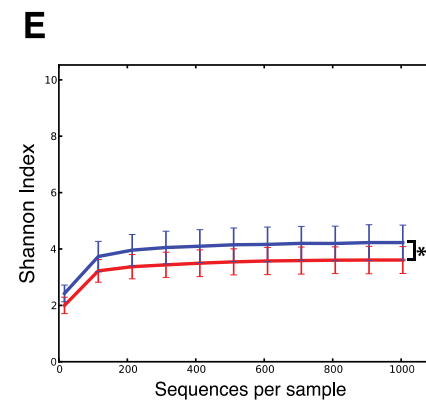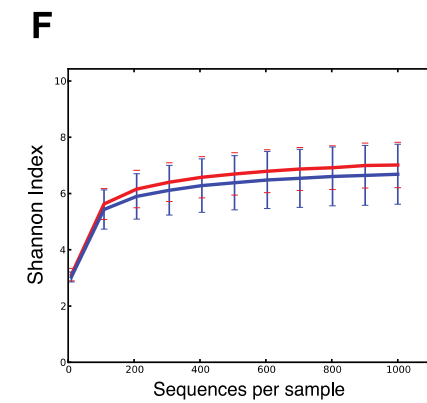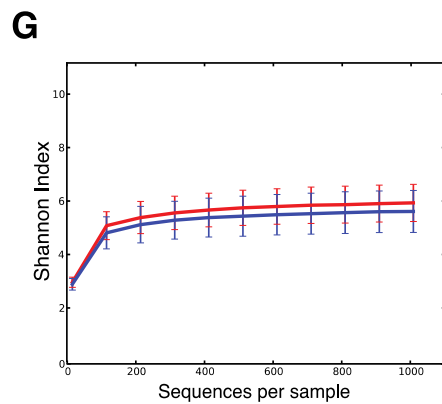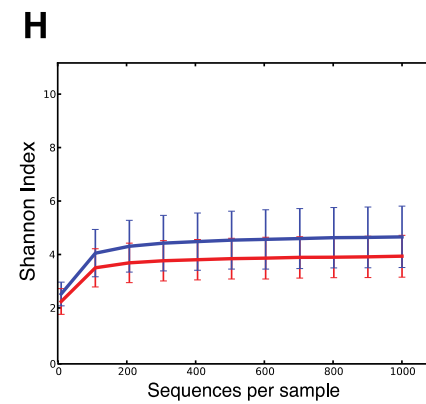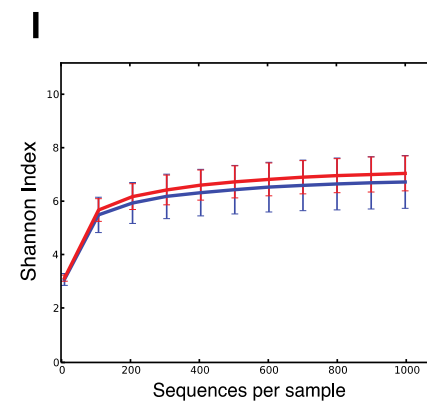

Supplement: S2 Fig — Rarefaction curves for fecal, oral, and skin samples at 1000 sequences per sample are shown, comparing (A-C) infected and control groups, (D-F) pre- and post-treatment groups, and (G-I) days 0 (blue) and 60 (red). Oral diversity significantly (*p<0.05, nonparametric t-test) decreased at the post-treatment time point in both control and infected groups). (PDF) [file pone.0212593.s003.pdf]

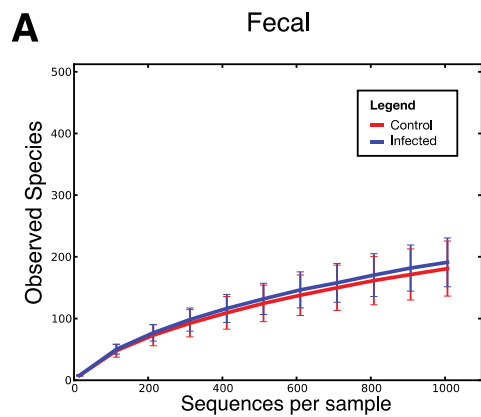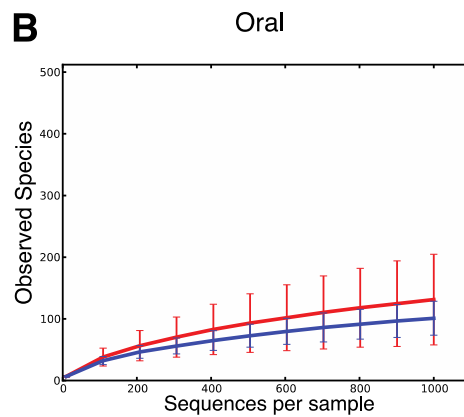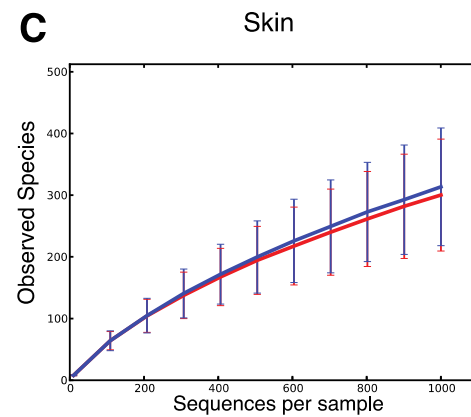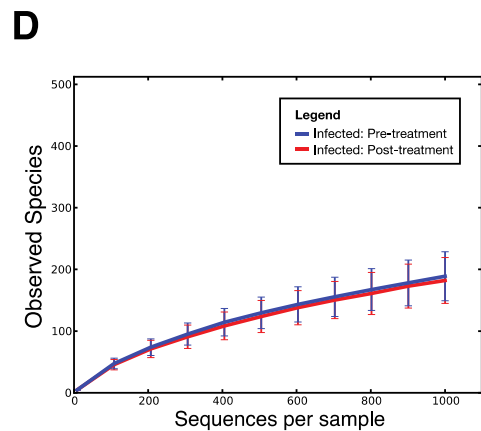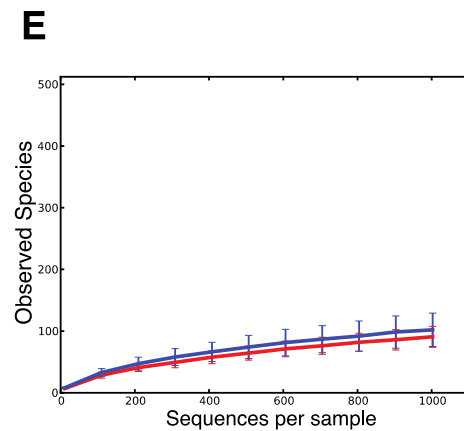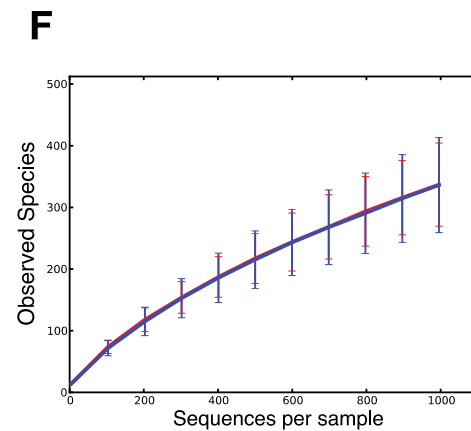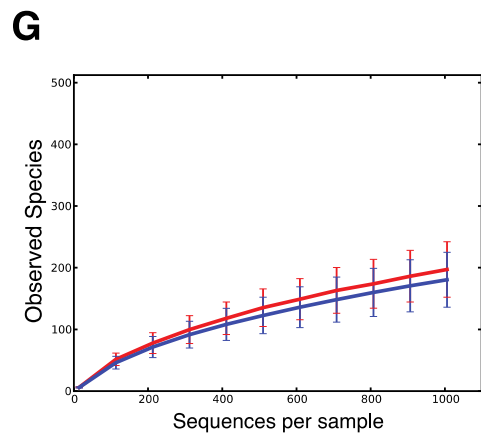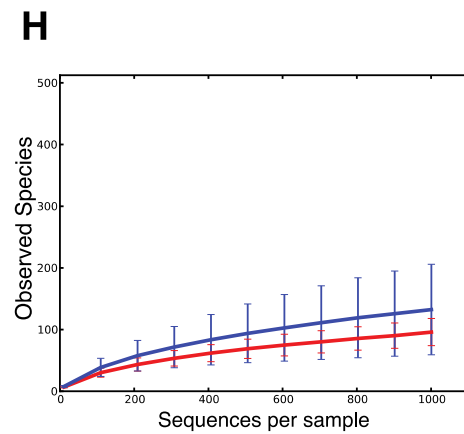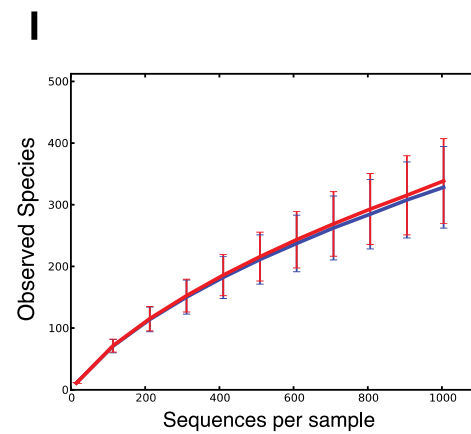

Supplement: S3 Fig — Rarefaction curves for fecal, oral, and skin samples based on the number of unique OTUs at 1000 sequences per sample are shown, comparing (A-C) Chagas infected and control groups, (D-F) pre- and post-treatment groups, and (G-I) days 0 (blue) and 60 (red). No significant (p < 0.05) differences between groups were found. (PDF) [file pone.0212593.s004.pdf]

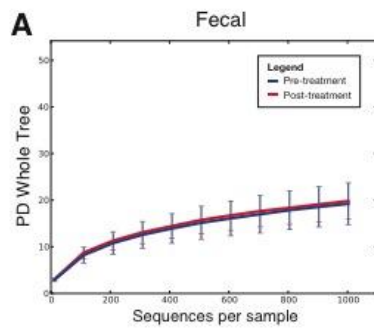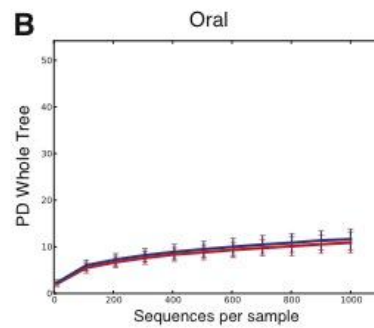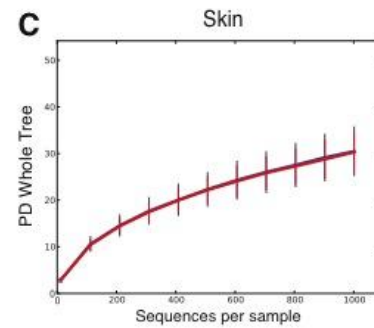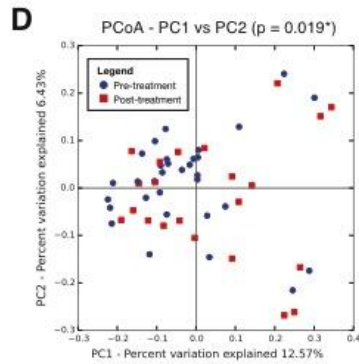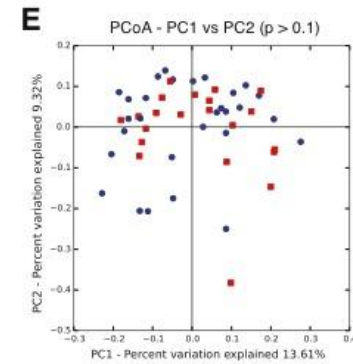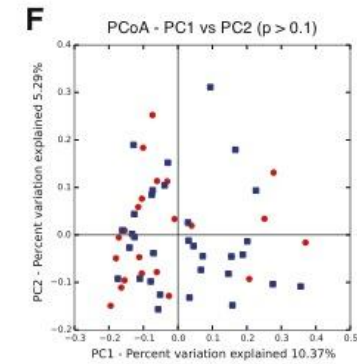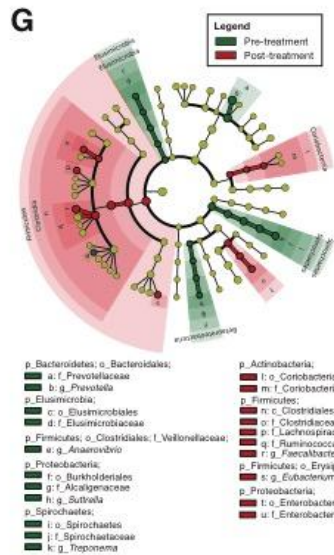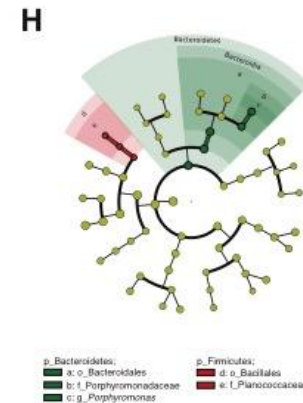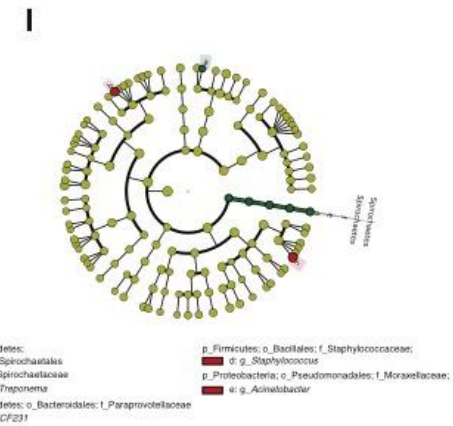

Supplement: S4 Fig — (A-C) Rarefaction curves for fecal, oral, and skin samples using Faith’s Phylogenetic Diversity (PD Whole Tree) metric at 1000 sequences per sample. (D-F) PCoA plots for fecal, oral, and skin samples using the unweighted UniFrac metric. (G-I) Taxa that best differentiate between children at the time points before (n = 32 for fecal and skin, and 29 for oral) and after treatment (n = 22 for fecal and skin, 21 for oral) found using using LEfSe using an LDA score cutoff of 3.0 and a minimum mean abundance of 0.1%. (PDF) [file pone.0212593.s005.pdf]
